# Supplementary material for: A temperature-adaptive component-dynamic-coordinated strategy for high-performance elastic conductive fibers
Source: Nat Commun. 2025 Jul 23;16:6785. doi: 10.1038/s41467-025-62140-y (PMC12287416; doi:10.1038/s41467-025-62140-y)
Supplement: Supplementary file 1 — Supplementary Information [file 41467_2025_62140_MOESM1_ESM.pdf]

Supplementary Information

A temperature-adaptive component-dynamic-coordinated strategy for high-performance elastic conductive fibers

Yue Zhang[1], Zechang Ming[2], Zijie Zhou[1], Xiaojie Wei[2], Jingjing Huang[2], Yufan Zhang[3], Weikang Li[1], Liming Zhu[1], Shuang Wang[1], Mengjie Wu[1], Zeren Lu[2], Xinran Zhou[3], Jiaqing Xiong[1, 3*]

[1]State Key Laboratory of Advanced Fiber Materials, College of Textiles, Donghua University, Shanghai 201620, China

[2]College of Materials Science and Engineering, Donghua University, Shanghai 201620, China

[3]Innovation Center for Textile Science and Technology, Donghua University, Shanghai 201620, China

*Corresponding author: jqxiong@dhu.edu.cn

The PDF file includes

Supplementary Notes 1 to 5

Supplementary Figures 1 to 39

Supplementary Tables 1 to 4

Supplementary References

Supplementary Notes:

Supplementary Note 1: Conductivity calculation for fiber and film samples

The conductivities of all fibers (thermoplastic polyurethane (TPU)/liquid metal microspheres (LMMS) (PUL), TPU/silver flakes (AgFK) (PUA), and TPU/AgFK/LMMS (PUAL)) and films (PUA and PUAL) follow the formula $G = L/RS$, where G represents the conductivity, L refers to the length of the test sample, R refers to the resistance of the corresponding length of the sample, and S refers to the cross-sectional area.

Supplementary Note 2: Fabrication of LMMSs with different sizes

Add 2 grams of gallium-indium liquid metal and 10 mL of N, N-dimethylformamide (DMF) solution into a centrifuge tube. Use a high-speed disperser to shear the liquid metal at 25,000 r min^{-1} for 10 minutes to obtain LMMSs with a median particle size of 40 μm. To obtain LMMSs with a median particle size of 20 μm, extend the shearing time to 20 minutes and reduce the shear speed to 18,000 r min^{-1}.

Supplementary Note 3: Preparation of PUA and PUAL cast films

PUA and PUAL solutions were first prepared by the methods and formulations shown in the ***Methods*** Section of the main content. The solutions were then poured into customized polytetrafluoroetylene (PTFE) molds for curing at 25 °C for 24 hours, to obtain cast films with dimensions of 2 cm × 1 mm × 1 mm, respectively.

Supplementary Note 4: Fabrication of styrene-isoprene-styrene (SIS)/AgFKs/LMMS fiber and poly(styrene-ethylene-butylene-styrene) (SEBS)/AgFKs/LMMS fiber

SIS or SEBS solution (25 wt%) was fabricated by adding SIS or SEBS particles into toluene under mechanical stirring at 60 °C for 2 h. Then, the SIS/AgFKs and SEBS/AgFKs spinning solutions were prepared at room temperature by adding AgFKs to the elastomer solution until evenly dispersed, where the mass ratio of AgFKs to SIS or SEBS was 3.5. Then LMMSs (the median particle size is 40 μm) were incorporated until homogeneous dispersion to fabricate the SIS/AgFKs/LMMS fiber and SEBS AgFKs/LMMS fiber through wet-spinning, where the mass ratio of LMMSs to TPU was 7. After standing to defoam, the spinning solution was injected via a needle (inner diameter of 510 μm) in the coagulation bath of water, in which the flow rate was regulated by syringe pumps at 20 mL h^{-1} and a winding collector was employed to collect the SIS/AgFKs/LMMS fiber and SEBS/AgFKs/LMMS fiber.

Supplementary Note 5: Demonstration of PUAL fiber's application in high-temperature perceptive firefighter suits

The high-temperature warning system consists of a main circuit and two branch circuits (**Supplementary Fig. 39**). An 8.5 V direct current (DC) power source is located in the main circuit, while branch circuit 1 is a series connection of a stretched (dynamic) PUAL fiber, and a red alarming light-emitting diode (LED), which are used to qualitatively judge whether the environment is secure or not. Tributary 2 is similar to tributary 1, which consists of an unstretched (static) PUAL fiber (~50 Ω) and a resistor (R0, ~200 Ω) in series for quantitative temperature identification.

The dynamic PUAL fiber attached to the arm produces variable resistance and partial voltage as a result of stretching and high-temperature activation. Wherein, under the same magnitude of stretching, the resistance, voltage, and resistance variation of the fiber under flame are lower compared to the case at room temperature. The temperature threshold is defined to be the lowest threshold of burn injury (50 ºC for 10 minutes)[1]. When the internal temperature of the firefighter suit reaches 50 °C, the surface temperature is measured to be 100 °C (**Supplementary Fig. 39**). Therefore, when the PUAL fiber's temperature reaches around 100 °C, a threshold partial voltage of the fiber was determined to be 0.8 V (≥ 0.8V, secure; < 0.8V, dangerous). Meanwhile, a signal processing module connected to the fiber determines the voltage value and enables a real-time safety display through the Bluetooth APP or on the laptop, indicating whether the firefighter is in a secure or dangerous state (**Fig. 6m, n** and **Supplementary Fig. 39**). Meanwhile, the alarming LED assigned to higher voltages on the fireproof suit becomes brighter and brighter, also indicating firefighters are approaching a high-temperature environment. Besides, in both room and high-temperature environments, the periodic voltage signals of the stretched fiber can also reflect the firefighter's motion state. Theoretically, the firefighter's movements could be determined in real-time through voltage waveform analysis, such as with the assistance of machine learning techniques.

At the same time, the static PUAL fiber approaching the high-temperature condition shows a significantly decreased resistance due to the heating-induced conductivity enhancement mechanism, which affects the voltage dispensation. By recording the voltage at both ends of the fiber and the fiber's temperature in real-time, a quantified relationship can be built to indicate the environmental temperature via the voltage (**Supplementary Table 4**). The real-time temperature information around the firefighter can be displayed through the Bluetooth APP on a mobile phone or a laptop, reminding both the firefighters and the staff at the monitoring station to take care or determine the safety status of the firefighter for taking appropriate measures (**Fig. 6m**, **n** and **Supplementary Fig. 39**), improving the safety in high-temperature operation.

Supplementary Figures

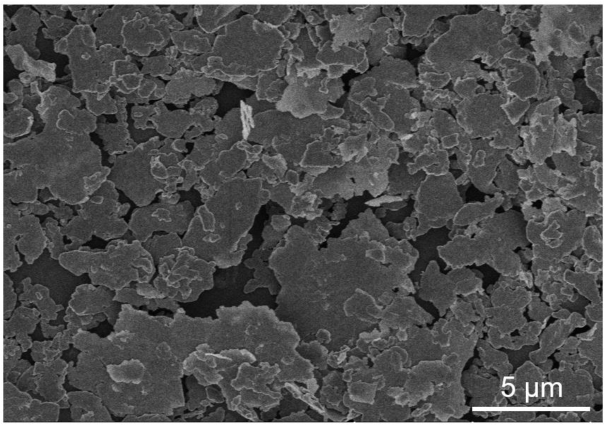

Supplementary Figure 1. Scanning electron microscope (SEM) image of AgFKs.

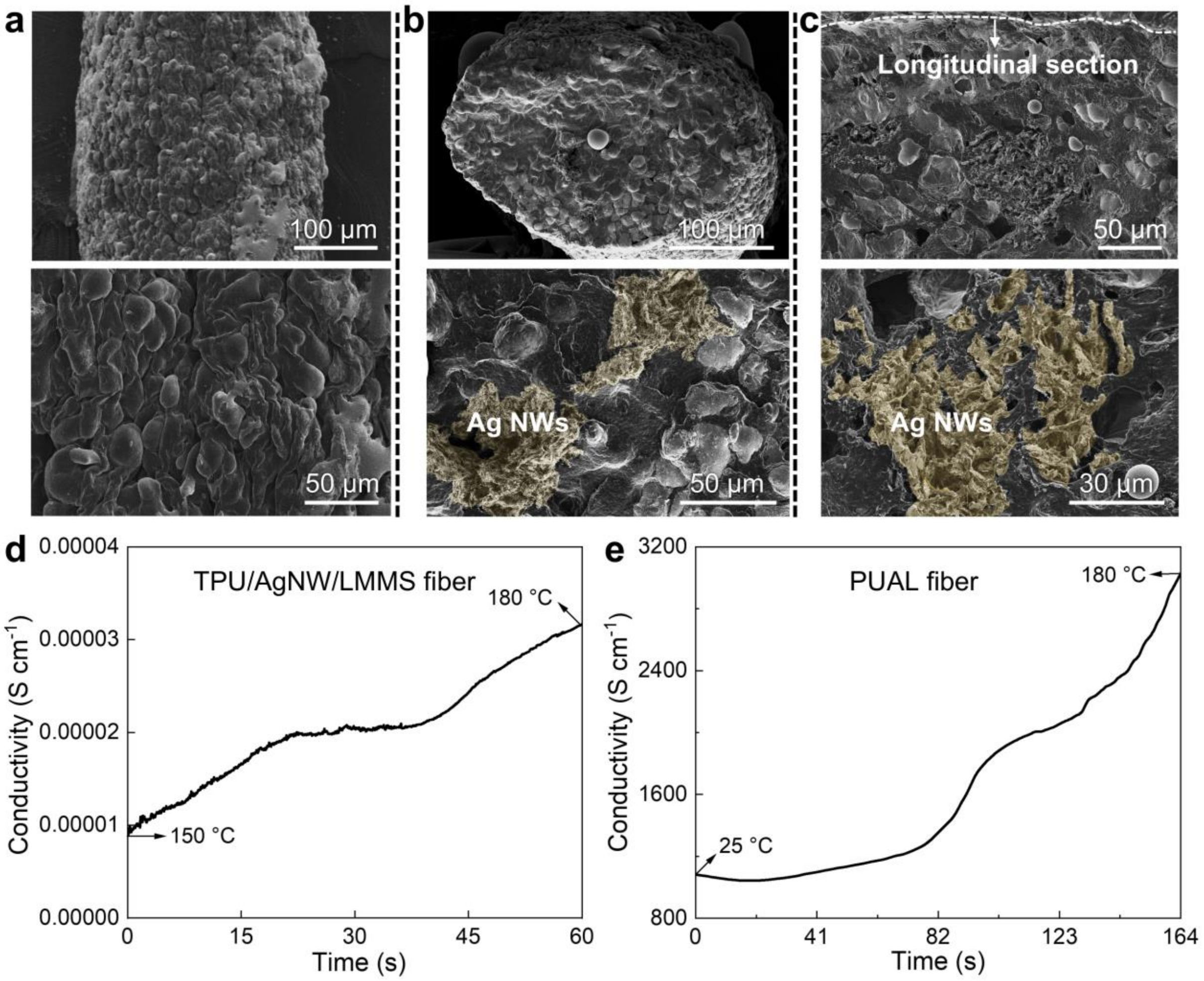

Supplementary Figure 2. SEM images and conductivity variation of TPU/silver nanowire (AgNW)/LMMS and PUAL fibers. **a**, Surface images. **b**, Cross-section images. **c**, Longitudinal-section images. **d**, Conductivity variation of TPU/AgNW/LMMS fiber from 150 °C to 180 °C, where the conductivity is undetectable below 150 °C. **e**, Conductivity variation of PUAL fiber from 25 °C (~1070 S cm^{-1}) to 180 °C (3020 S cm^{-1}). We choose AgFKs instead AgNWs for elastic conductive fiber (ECF) fabrication is because equivalent quality AgFKs are much cheaper than AgNWs[2] (AgFK: $1.4/g, AgNW: $550/g), and the PUAL fibers show much higher conductivity than the AgNW-based fibers, where AgNW-based fibers' conductivity is too small to be detectable from 25 °C to 150 °C, and reaches only ~3×10^{-5} S cm^{-1} when heated to 180 °C.

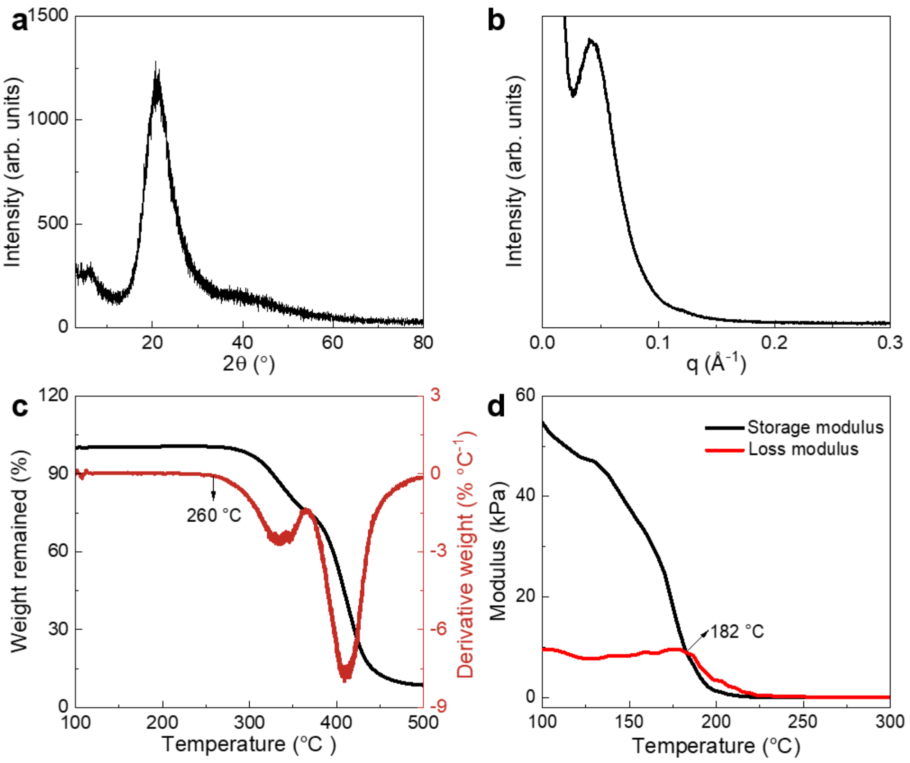

Supplementary Figure 3. Thermodynamic properties of TPU fiber. **a**, X-ray diffraction (XRD) patterns of the fiber. **b**, X-ray scattering (SAXS) curve of the fiber. **c**, Thermogravimetric analysis (TGA) curve of the fiber. **d**, Temperature-dependent rheological behaviors to verify the viscous state temperature of the TPU matrix.

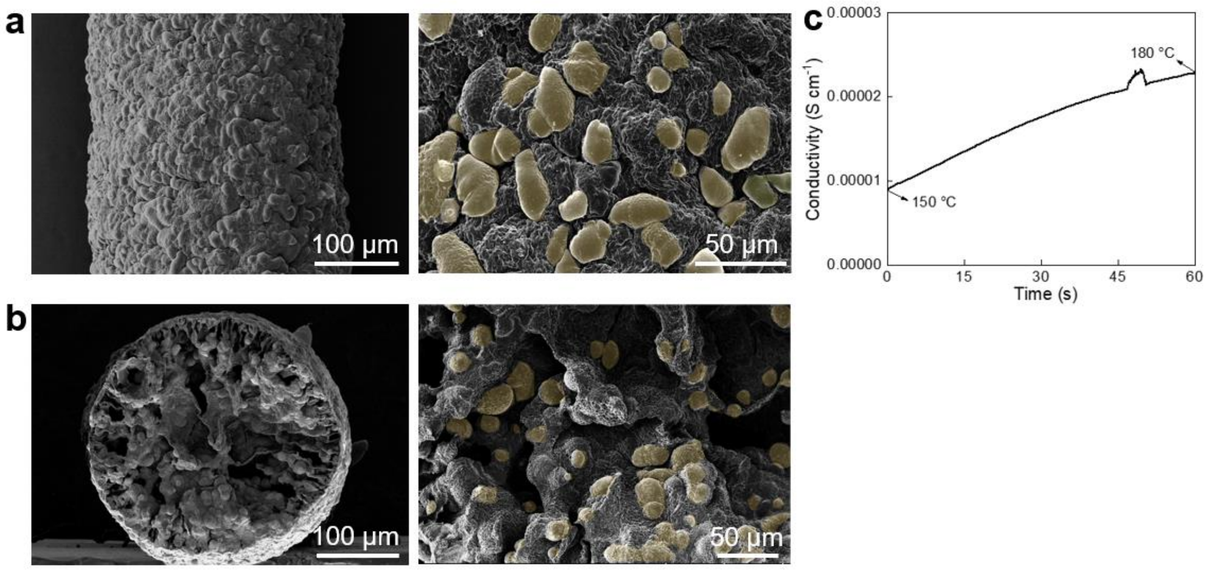

Supplementary Figure 4. Morphology and conductivity of PUL fibers (without AgFKs). a, Surface SEM image. **b**, Cross-sectional SEM images. **c**, Conductivity variation of the fiber when heated from 150 ℃ to 180 ℃. The main LMMSs in the enlarged SEM images of the fiber have been marked in bright yellow, which are wrapped and isolated by the TPU matrix both inside and on the surface, leading to discontinuous conductive pathways. Therefore, the fiber is nonconductive under ambient conditions (25 ℃). The conductivity is only measurable when the temperature rises above 150 ℃, which is increased from ~9×10^{-6} S cm^{-1} (150 ℃) to ~2×10^{-5} S cm^{-1} (180 ℃) as the temperature continuously rises, indicating that only at high temperatures the LMMS can be activated to deform/rupture and LMMSs make limited contributions on the fiber's conductivity enhancement. Thus, AgFKs play the key role in the PUAL fiber at either ambient conditions or high temperatures by forming a dominant conductive path.

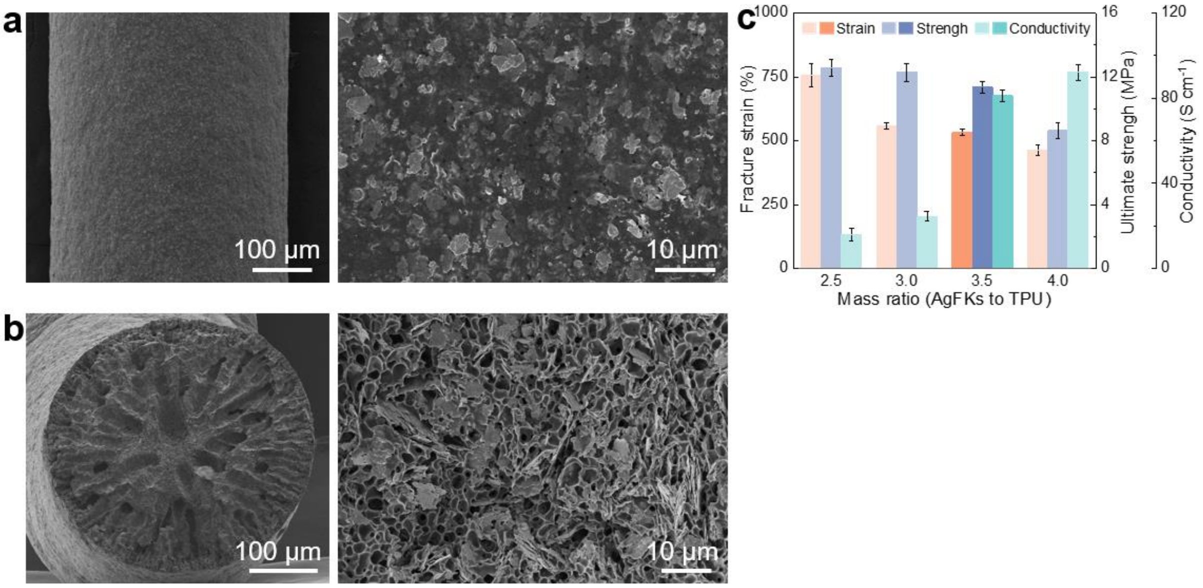

Supplementary Figure 5. Morphology, mechanical and conductivity variation of PUA fiber. a, Surface SEM images. **b**, Cross-sectional SEM images. **c**, Fracture strain, ultimate strength and conductivity of PUA fiber (five parallel samples were measured, and error bars represent the standard deviation of the mean) with different mass ratio of AgFKs to TPU. The PUA fiber's conductivity can be significantly enhanced (~230% increment) by improving the mass ratio of AgFKs to TPU from 3 to 3.5, without obvious scarification on mechanical properties (~7% decrease in ultimate strength and ~4% decrease in fracture strain). Therefore, the optimal mass ratio of AgFKs to TPU is confirmed to be 3.5.

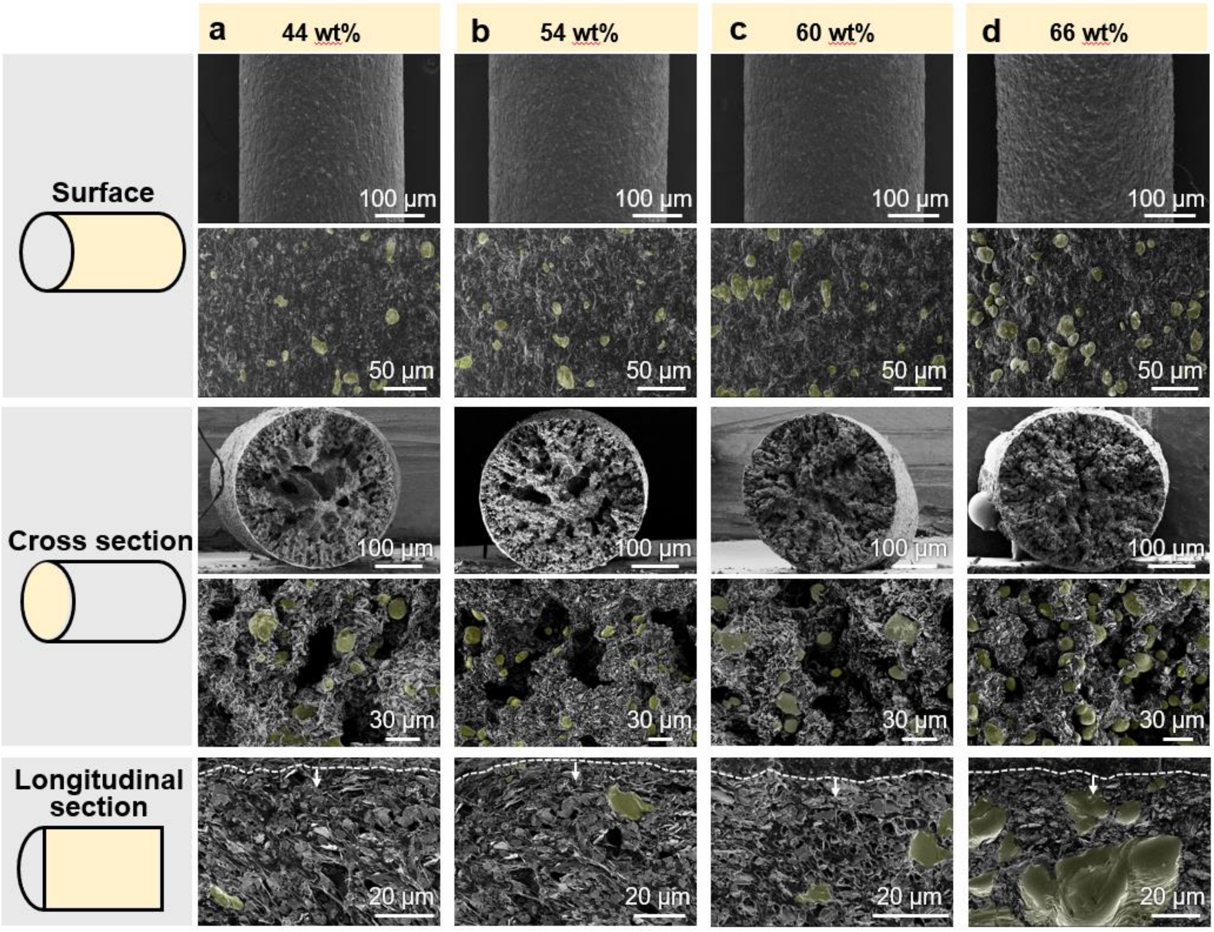

Supplementary Figure 6. SEM images of PUAL fibers prepared with different contents of LMMSs. **a**, 44 wt%. **b**, 54 wt%. **c**, 60 wt%. **d**, 66 wt%. The main LMMSs in the enlarged SEM images of the fibers have been marked in bright yellow. Too few LMMSs tend to be unevenly distributed and cannot form effective connections to the AgFKs. Too many LMMSs easily agglomerate due to their high surface tension, and some excess LMMSs are exposed on the fiber's surface. Both are not good for sufficient electrical connection in the fiber, resulting in relatively poor conductivity (**Supplementary Fig. 7f, h**).

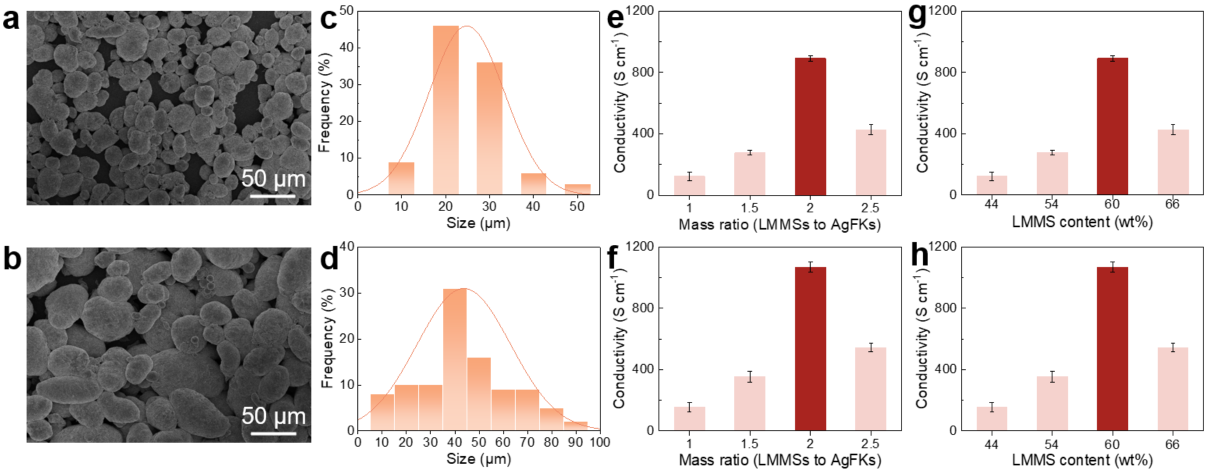

Supplementary Figure 7. Morphology and size distribution of LMMSs, and conductivity of PUAL fibers. a, **b**, SEM images of LMMSs with the median particle size to be (**a**) 20 μm and (**b**) 40 μm. **c**, **d**, Size distribution of LMMS with the median particle size to be (**c**) 20 μm and (**d**) 40 μm. Conductivity of $PUAL_{20}$ fibers and PUAL fibers with (**e**, **f**) different mass ratios of LMMSs to AgFKs and (**g**, **h**) different contents of LMMSs. Five parallel samples were measured, and error bars represent the standard deviation of the mean for **e**–**h**. When the mass ratio of LMMSs to AgFKs is 1, 1.5, 2, and 2.5, the corresponding LMMS mass fraction of the whole system is 44 wt%, 54 wt%, 60 wt%, and 66 wt%. To sum up, the optimal mass ratio of LMMSs to AgFKs is 2 (60 wt% LMMS in the whole system), rendering the optimum electrical conductivity of the fiber.

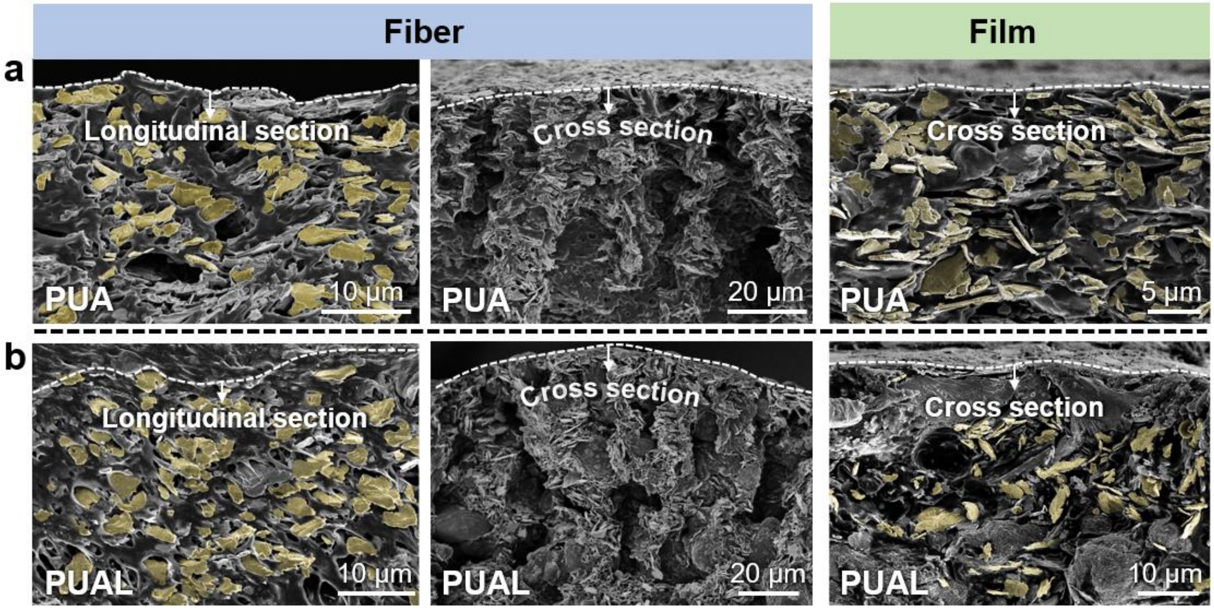

Supplementary Figure 8. SEM images of PUA and PUAL fibers and cast films with the same compositions. a, PUA fiber and cast film. **b**, PUAL fiber and cast film. From left to right are the fiber axial longitudinal-section, fiber cross-section, and film cross-section of the samples. The main AgFKs in the axial longitudinal-section SEM image of the fiber and the cross-section SEM image of the film are marked in bright yellow.

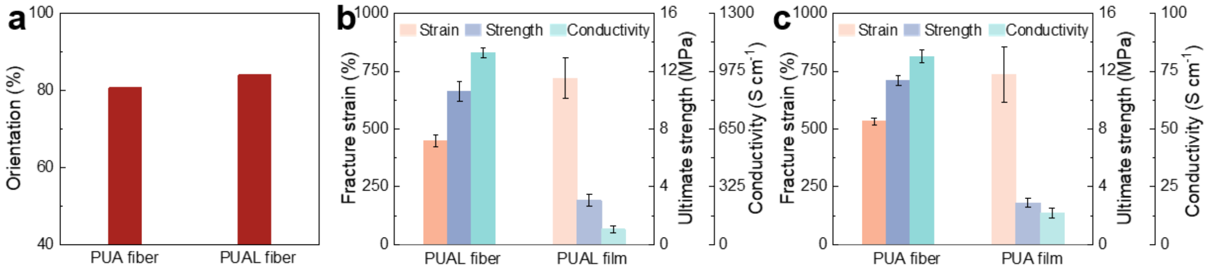

Supplementary Figure 9. Orientation, conductivity, and mechanical properties of PUA and PUAL fibers, and film samples. a, Axial orientation of AgFKs within PUA and PUAL fibers, calculated from the analysis of SEM images. About 50 AgFKs were randomly taken from the longitudinal-section SEM images to measure the angle between their long axis direction and the fiber's axial direction, where the angle less than 45 degrees was counted as oriented and the ratio of oriented AgFKs was calculated. **b**, **c**, Mechanical and electrical properties of (**b**) PUAL and (**c**) PUA fibers compared with the films with the same compositions. Five parallel samples were measured, and error bars represent the standard deviation of the mean for **b, c.**

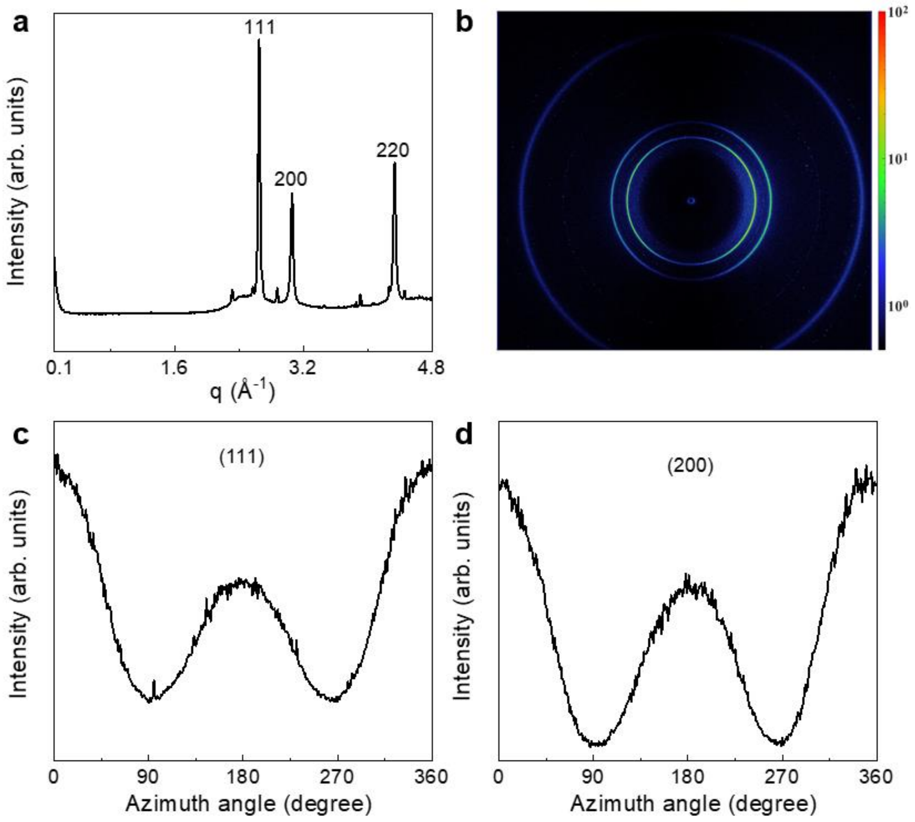

Supplementary Figure 10. Wide-angle x-ray scattering (WAXS) of PUAL fiber. a, WAXS curves of PUAL fiber. **b**, 2D WAXS pattern. **c**, The azimuthal plot of scattering at (111) along the φ direction in the region of 0° to 360°. **d**, The azimuthal plot of scattering at (200) along the φ direction in the region of 0° to 360°. Three obvious diffraction peaks corresponding to the lattice plane of (111), (200) and (220) were observed in the WAXS profiles of PUAL fiber[3]. Furthermore, Hermann orientation factor (*f*) can be calculated according to the following equations[4].

$$f = \frac{3<\cos^2 \varphi>-1}{2} \quad (1)$$

$$< \cos^2 \varphi > = \frac{\int_0^{2\pi} I(\varphi)\sin\varphi\cos^2\varphi\, d\varphi}{\int_0^{2\pi} I(\varphi)\sin\varphi\, d\varphi} \quad (2)$$

The value of *f* is between 0 (for random orientation) and 1 (for a perfectly oriented sample). *I*(φ) is the 1D intensity distribution along with the azimuthal angle φ of the (111) and (200) planes of AgFKs (**c**, **d**). In this paper, the orientation factor *f* of PUAL fiber was calculated to be 0.25, indicating a certain degree of orientation existed in the fiber. The relatively low orientation factor is because AgFKs align well along the axial direction but have varied orientations along the radial direction. This anisotropic orientation cannot be well reflected in WAXS but the WAXS result can be regarded as supplementary evidence, and the directly calculated orientation from the SEM images would be more intuitive and convincing to reflect the AgFKs' orientation level in fiber samples.

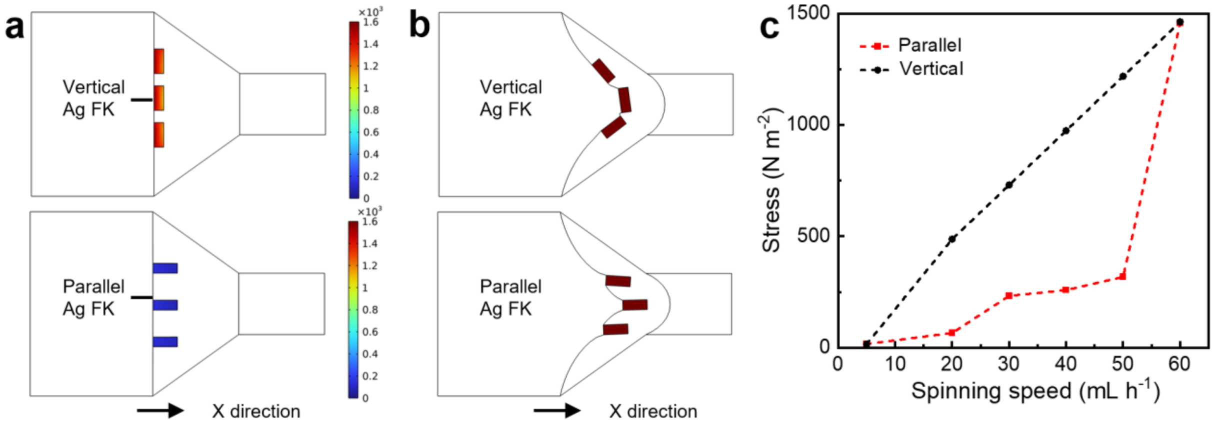

Supplementary Figure 11. Hydrodynamic analysis and orientation calculation of AgFKs under different wet-spinning speeds. a, Stress diagram of AgFKs in X direction (wet-spinning flow direction) at a flow rate of 20 mL h^{-1} in the initial state. **b**, Flow-induced orienting AgFKs at a constant time scale at a flow rate of 20 mL h^{-1}. **c**, Maximum AgFKs stress under different wet-spinning speeds in the initial state. The simulation results show that AgFK's orientation is closely related to the stresses induced by the shear effect at the syringe funnel (**a**). The stresses on AgFK parallel to the flow direction are significantly lower than those in the perpendicular direction (the cloud diagrams are processed in absolute value for ease of presentation), causing AgFKs in the perpendicular direction of the flow are prone to rotate, while those in the parallel direction of the flow are relatively stable (**b**). Therefore, AgFKs in the perpendicular flow direction tend to orient parallel to the flow direction. Such an orientation can be attributed to the stress difference acting on the AgFK, which is directly related to the applied inlet velocity which has the same physical meaning as the wet-spinning speed. The transformation formula for the two is as follows.

$$v_{inlet} = \frac{3600\, v_{wet\ spinning}}{\pi r^2} \quad (3)$$

As shown in (**c**), a systematic study of wet-spinning speed (from 5 mL h^{-1} to 60 mL h^{-1}) in the initial state reveals obvious stress differences in vertical and parallel can be produced with a spinning speed from 20 mL h^{-1} to 50 mL h^{-1}, which could be a driving force to improve the AgFKs orientation. The above simulation results aid in illustrating that the flow-induced shearing force in wet-spinning leads to the high orientation of AgFKs along the fiber's axial direction.

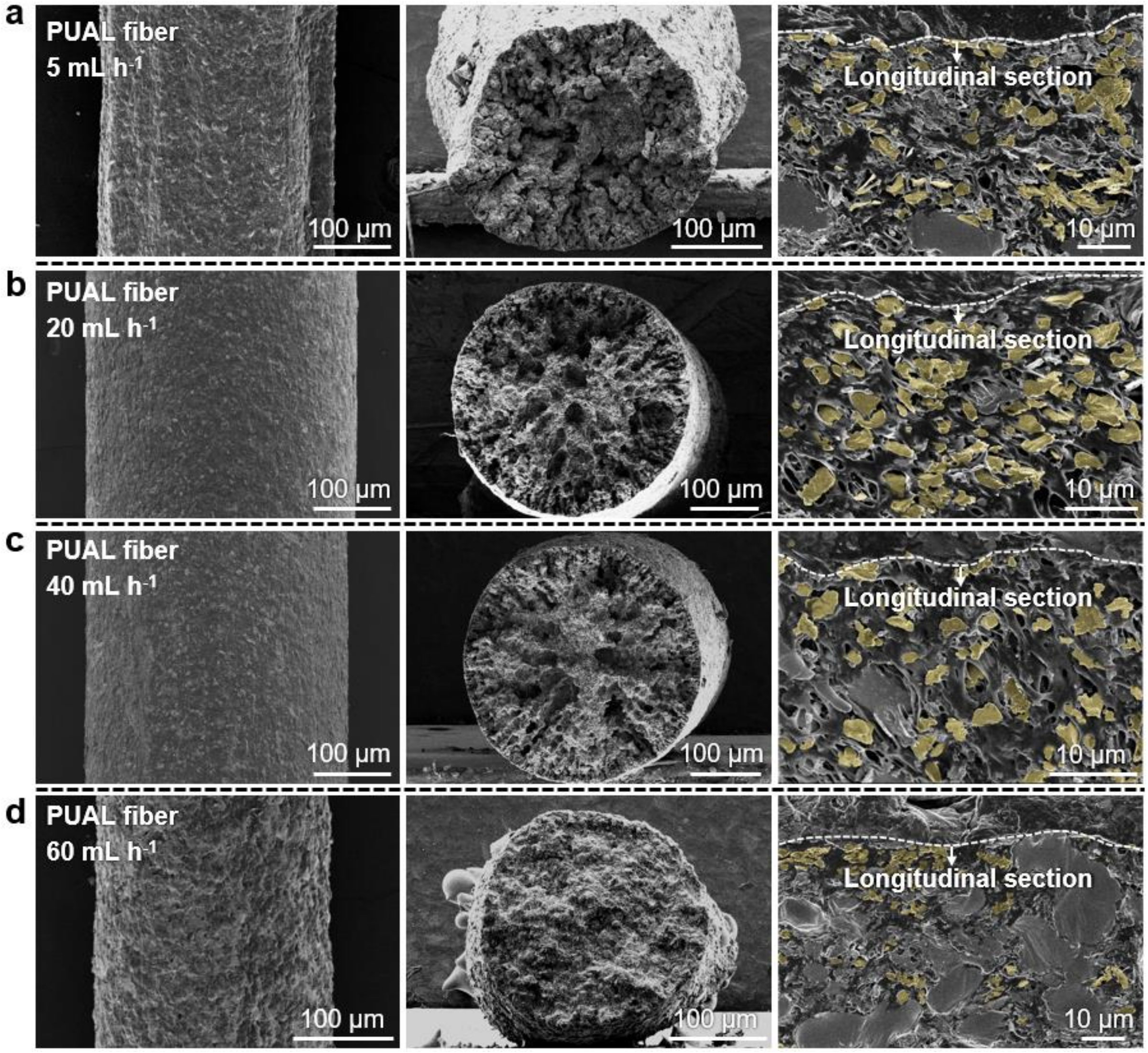

Supplementary Figure 12. SEM images of PUAL fibers prepared with different wet-spinning speeds. **a**, 5 mL h^{-1}. **b**, 20 mL h^{-1}. **c**, 40 mL h^{-1}. **d**, 60 mL h^{-1}. The AgFKs in the enlarged images have been marked in yellow. The cross-sectional morphology suggests that the flow rate affects both fiber uniformity and filler orientation. When the spinning speed is too slow (e.g., 5 mL h^{-1}), AgFKs have a relatively lower orientation since the small speed gradient (between the ink inside the wide syringe and the thin needle) renders a small shear rate, leading to irregular cross-sections of the fibers (**a**). However, a too-fast speed (e.g., 60 mL h^{-1}) will cause turbulent flow, which increases the stress difference between the edge and the center of the solution flow, disturbing the filler orientation and resulting in fibers with a non-uniform diameter (**d**)[5]. In comparison, suitable wet-spinning speeds (e.g., 20 mL h^{-1} and 40 mL h^{-1}) form fibers with a circular cross-section (**b**, **c**).

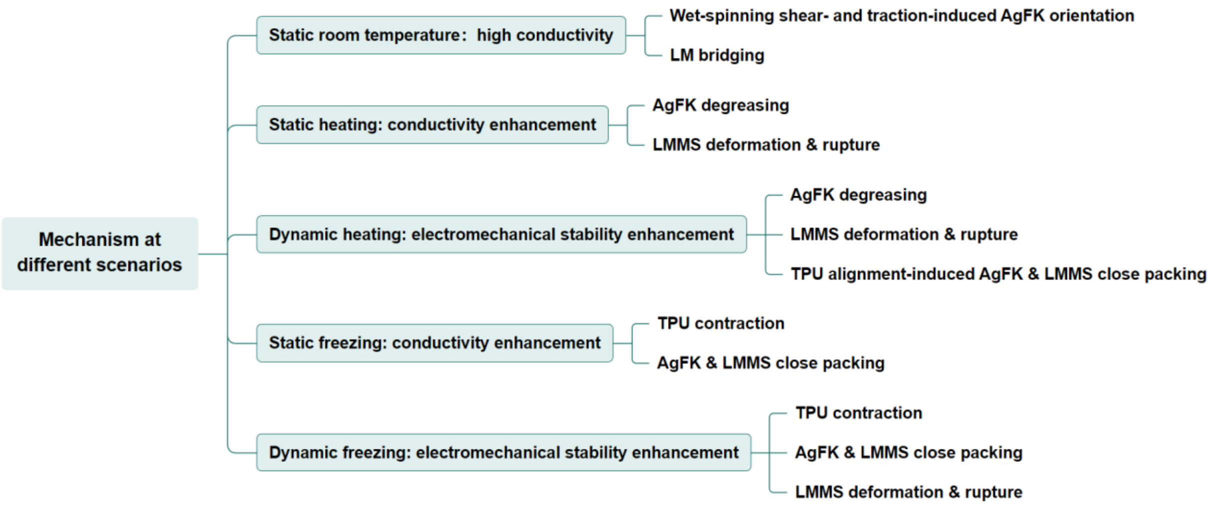

Supplementary Figure 13. Summary of applicability scopes of different mechanisms.

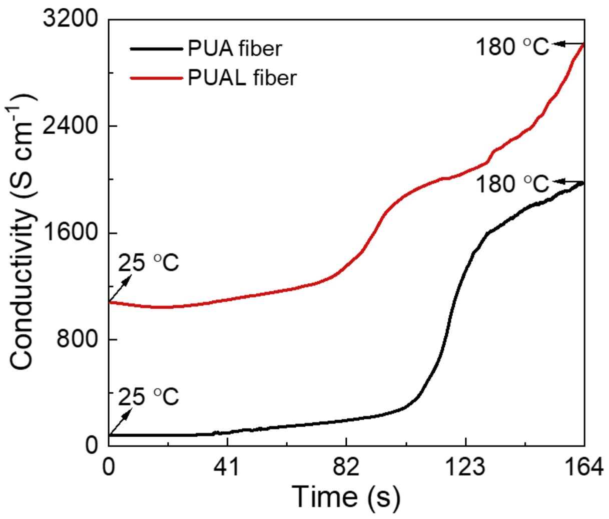

Supplementary Figure 14. Conductivity variation of the PUA and PUAL fibers when heated from 25 ℃ to 180 ℃.

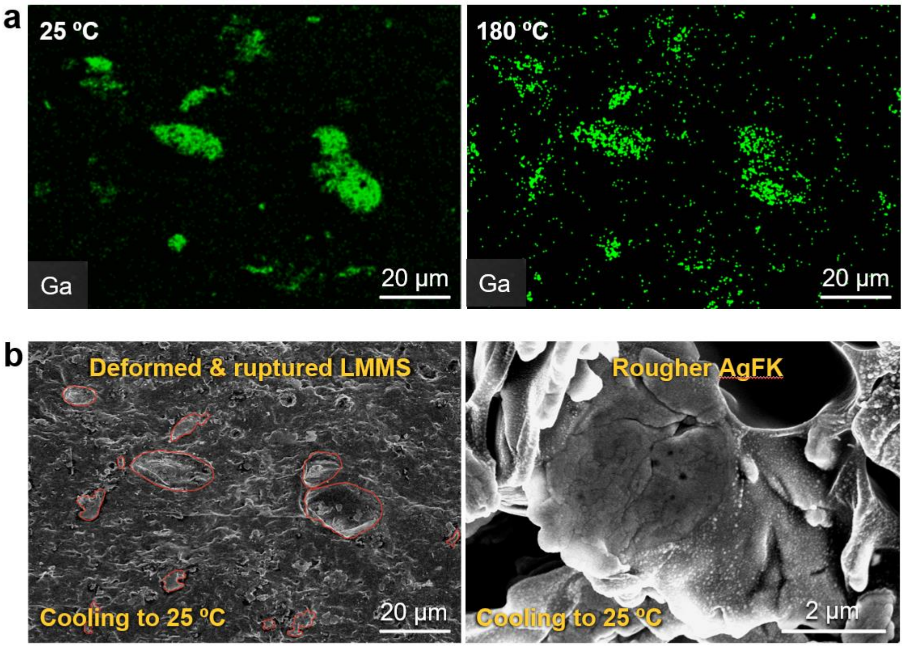

Supplementary Figure 15. SEM images and energy dispersive spectroscopy (EDS) mapping of LMMSs and AgFKs at different temperatures. a, EDS mapping of LMMS of PUAL fiber at 25 °C and 180 °C. **b**, SEM image of deformed and ruptured LMMSs and roughened AgFKs upon cooling from 180 °C to 25 °C. These indicate the irreversible thermal-induced morphology transformation that ensures a reliable heating-induced conductivity enhancement mechanism. The main deformed and ruptured LMMSs of the fiber after the cooling process have been marked by red circles.

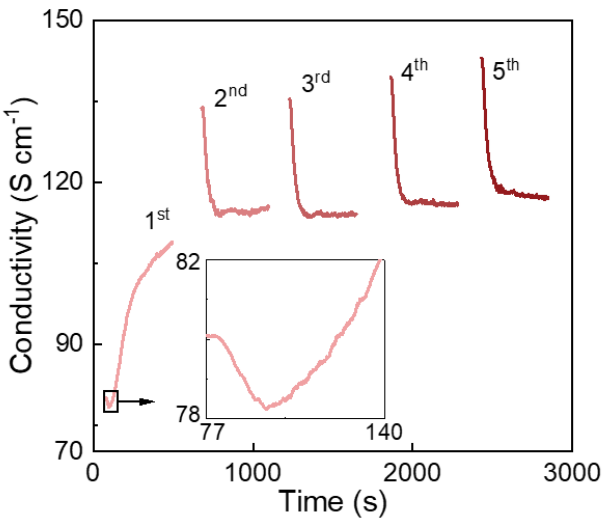

Supplementary Figure 16. Conductivity variation of the PUA fiber when cyclically heated from 25 °C to 100 °C with natural cooling for each cycle (the start and end points of each curve correspond to the fiber's conductivity at 25 °C and 100 °C, respectively, with a two-hour interval between each cycle).

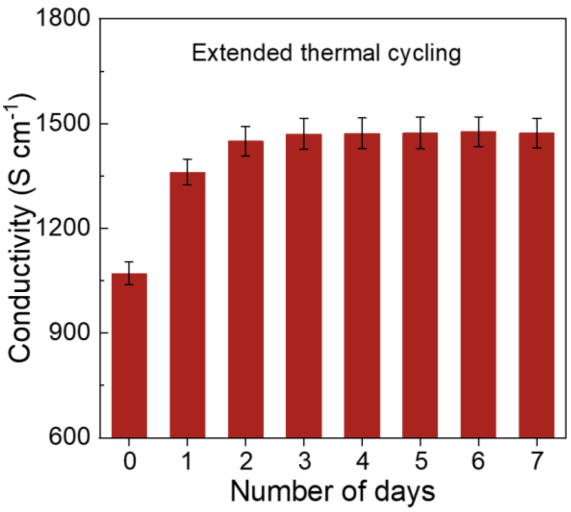

Supplementary Figure 17. The PUAL fiber's conductivity variation during multi-cyclic heating (heating from 25 °C to 100 °C, one cycle every day) over 7 days (five parallel samples were measured, and error bars represent the standard deviation of the mean). PUAL fibers' conductivity shows a large increase (in the first cycle: from ~1070 S cm^{-1} (initial) to 1361 S cm^{-1} (after 1 heating cycle), then a small increase (to ~1450 S cm^{-1}) after 2 heating cycles, and to ~1470 S cm^{-1} after 3 heating cycles), and eventually remaining a highly stable value of ~1470 S cm^{-1} from 4 to 7 cycle. Since this is a static heating-induced conductivity enhancement process under 100 °C, the LMMS rupture does not function (see details in the explanation of **Supplementary Fig. 4**) and the reason for the conductivity increase is the increasing degreasing degree of AgFKs under elevated temperature. Therefore, the PUAL fiber presents a stable terminating conductivity when reaching the saturation of AgFK degreasing.

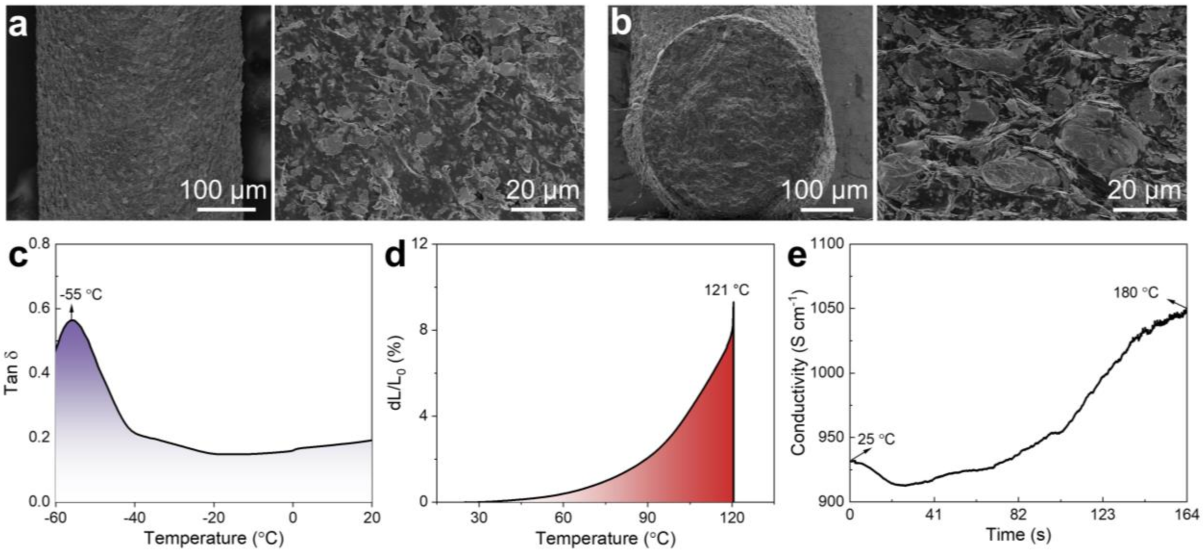

Supplementary Figure 18. Morphology, electrical conductivity, and thermodynamic properties of the SEBS/AgFKs/LMMS fiber. **a**, Surface SEM image. **b**, Cross-sectional SEM image. **c**, Temperature-dependent tan δ. **d**, Thermomechanical analysis (TMA) curve. **e**, Conductivity variation from 25 ℃ to 180 ℃. **c** and **d** indicate that the fiber' rubbery state temperature range is from -55 ℃ to 121 ℃.

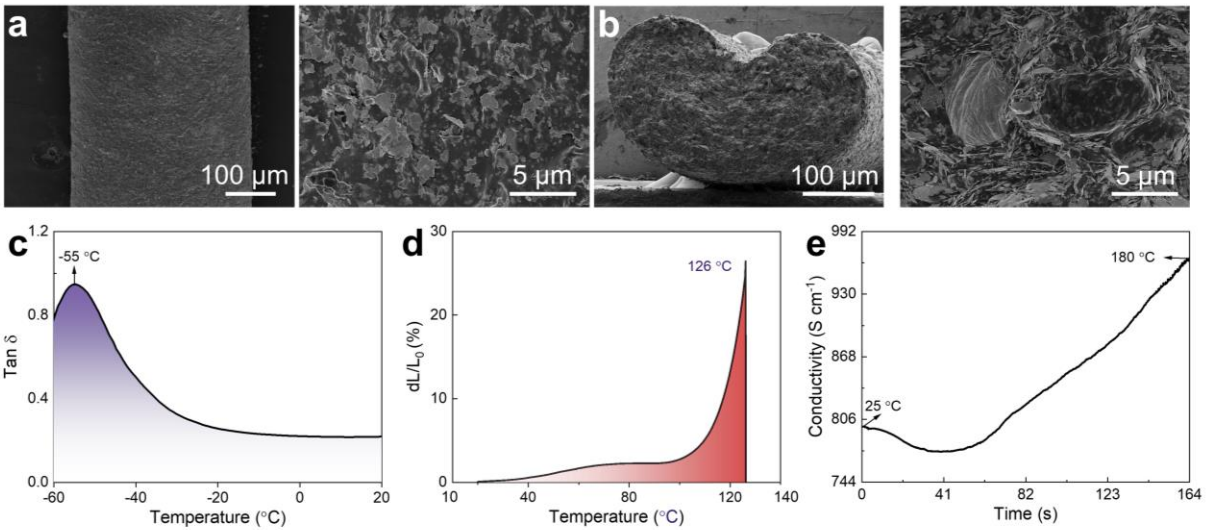

Supplementary Figure 19. Morphology, electrical conductivity, and thermodynamic properties of the SIS/AgFKs/LMMS fiber. a, Surface SEM image. **b,** Cross-sectional SEM image. **c**, Temperature-dependent tanδ. **d**, TMA curve. **e**, Conductivity variation from 25 ℃ to 180 ℃. **c** and **d** indicate that the fiber' rubbery state temperature range is from -55 ℃ to 126 ℃.

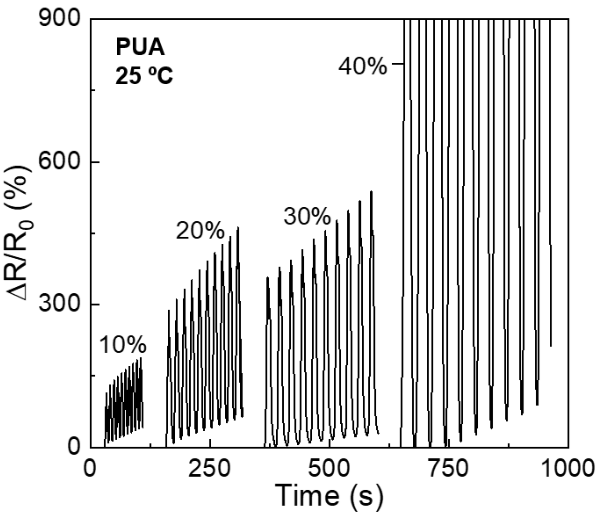

Supplementary Figure 20. Resistance change of PUA fiber under cyclic stretching with different strains (10%–40%) at 25 ℃.

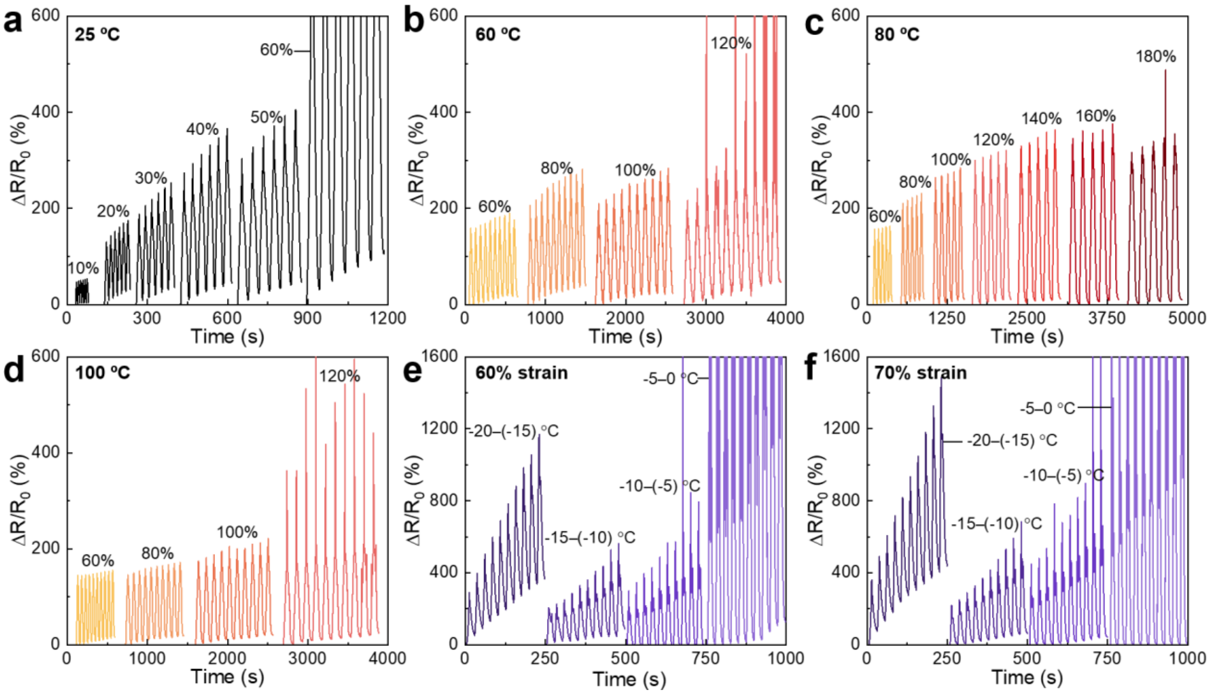

Supplementary Figure 21. Resistance change of the $PUAL_{20}$ fibers under cyclic stretching at different strains and temperatures. **a**, Cyclic stretching at 25 ℃ (10%–60% strain). **b**, Cyclic stretching at 60 ℃ (60%–120% strain). **c**, Cyclic stretching at 80 ℃ (60%–180% strain). **d**, Cyclic stretching at 100 ℃ (60%–120% strain). **e**, Cyclic stretching with 60% strain from -20 ℃ to 0 ℃. **f**, Cyclic stretching with 70% strain from -20 ℃ to 0 ℃. 20 cycles were tested for each condition, with the last 5–10 cycles plotted for clarity. Shades of purple from dark to light in **e, f** represent the temperature ranges of -20 °C to -15 °C, -15 °C to -10 °C, -10 °C to -5 °C, and -5 °C to 0 °C, respectively.

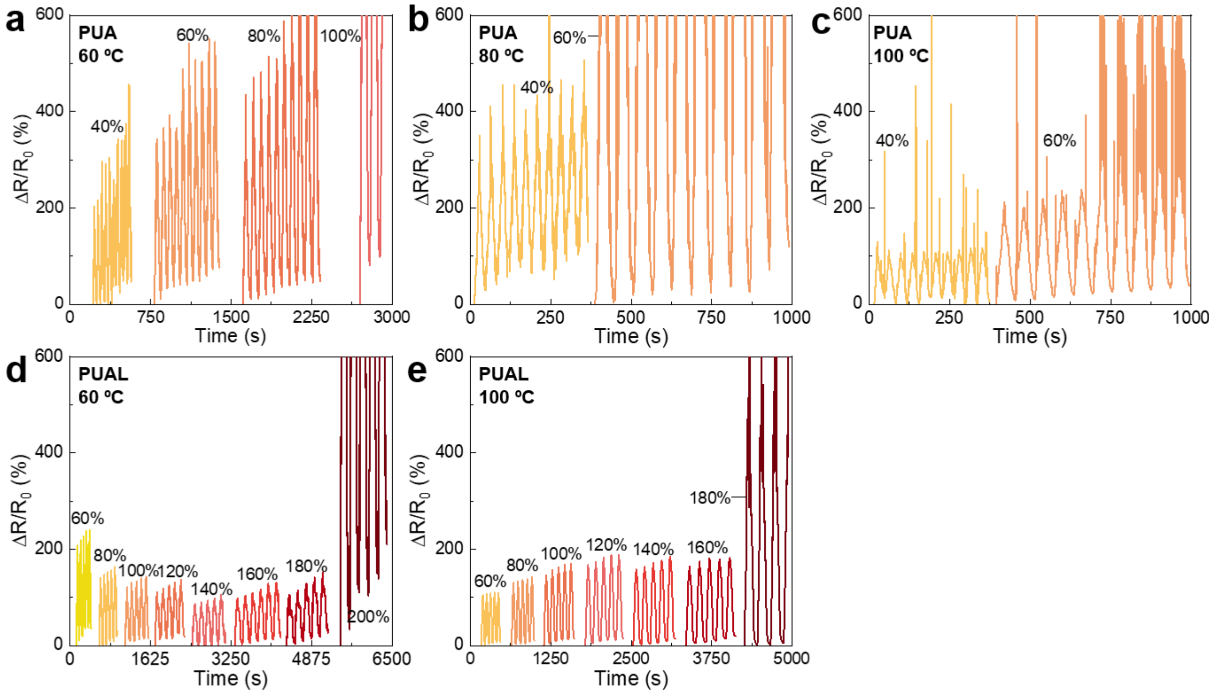

Supplementary Figure 22. Resistance change of PUA and PUAL fibers under cyclic stretching at different strains and temperatures. a, Cyclic stretching of PUA fiber at 60 °C (40%–100% strain). **b**, Cyclic stretching of PUA fiber at 80 °C (40%–60% strain). **c**, Cyclic stretching of PUA fiber at 100 °C (40%–60% strain). **d**, Cyclic stretching of PUAL fiber at 60 °C (60%–200% strain). **e**, Cyclic stretching of PUAL fiber at 100 °C (60%–180% strain). 20 cycles were tested for each condition, with the last 5–10 cycles plotted for clarity.

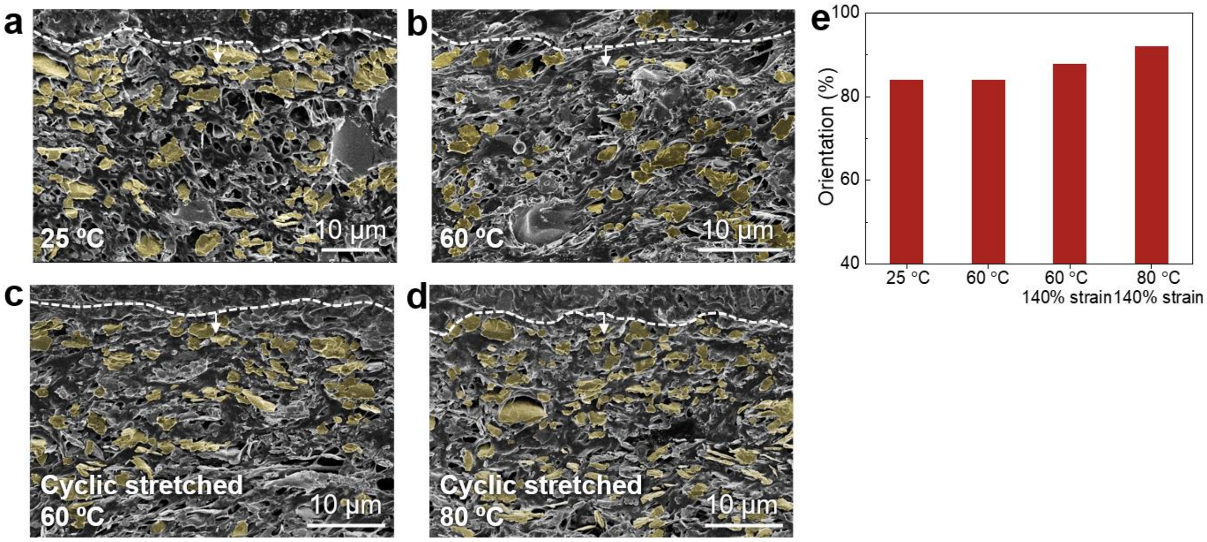

Supplementary Figure 23. Morphology and orientation degree of PUAL fibers at different temperatures with and without stretching. a–d, SEM image of the longitudinal section of PUAL fiber at (**a**) 25 °C without stretching, (**b**) heating at 60 °C for one hour, (**c**) stretching with 140% strain for 20 cycles at 60 °C and (**d**) stretching with 140% strain for 20 cycles at 80 °C. **e**, Orientation degree comparison of PUAL fibers obtained under the above four conditions. The main AgFKs in the fibers have been marked in bright yellow. Under the combined effect of cyclic stretching and heating, AgFKs' orientation is enhanced, achieving the highest orientation degree of 92% at 80 °C, 140% strain.

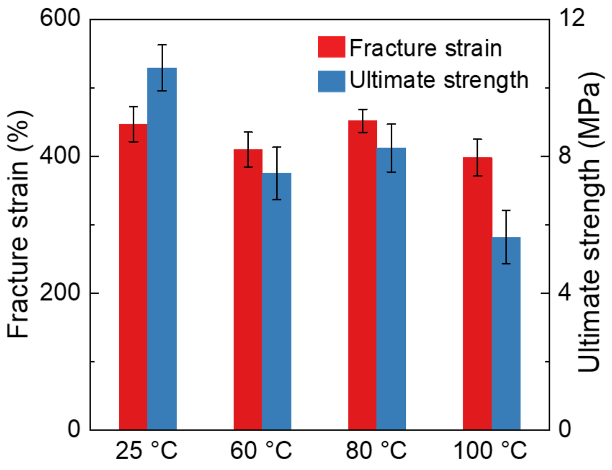

Supplementary Figure 24. Fracture strain and ultimate strength of the PUAL fiber at 25 ℃ and after being heated at 60 ℃, 80 ℃, and 100 ℃ for 30 minutes. Five parallel samples were measured, and error bars represent the standard deviation of the mean.

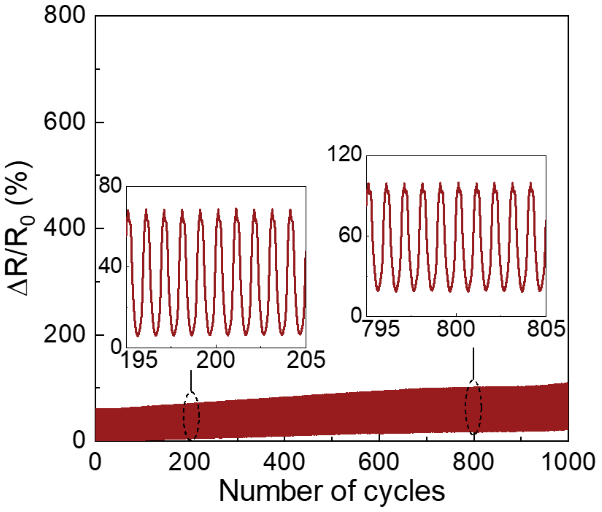

Supplementary Figure 25. Electrical stability of PUAL fiber with a thicker diameter of 1 mm at 80 °C under 60% strain over 1000 stretching cycles.

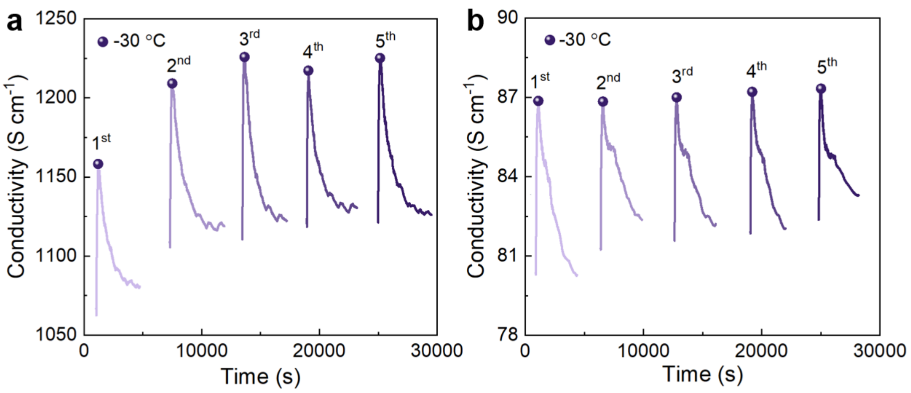

Supplementary Figure 26. Conductivity variation of (**a**) PUAL fiber and (**b**) PUA fiber in five freezing and warming cycles from 25 °C to -30 °C and to 25 °C. The start and end points of each curve correspond to the conductivity at 25 °C (initial) and 25 °C (final), respectively, with a two-hour interval between each cycle.

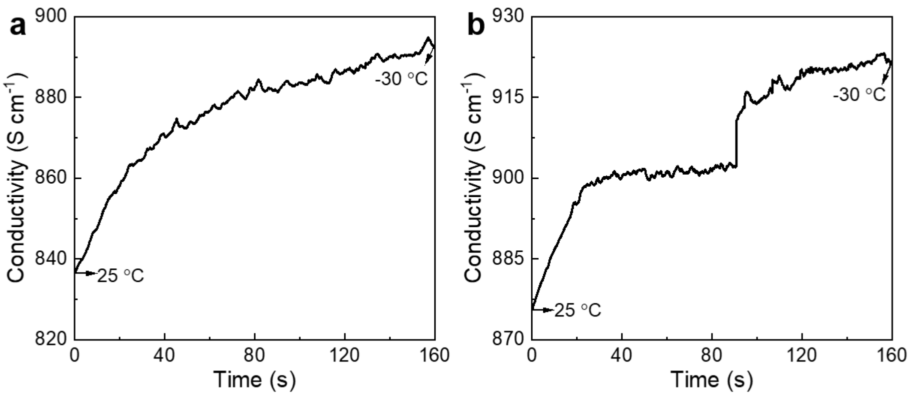

Supplementary Figure 27. Conductivity variation of (**a**) SEBS/AgFKs/LMMS fiber and (**b**) SIS/AgFKs/LMMS fiber from 25 ℃ to -30 ℃.

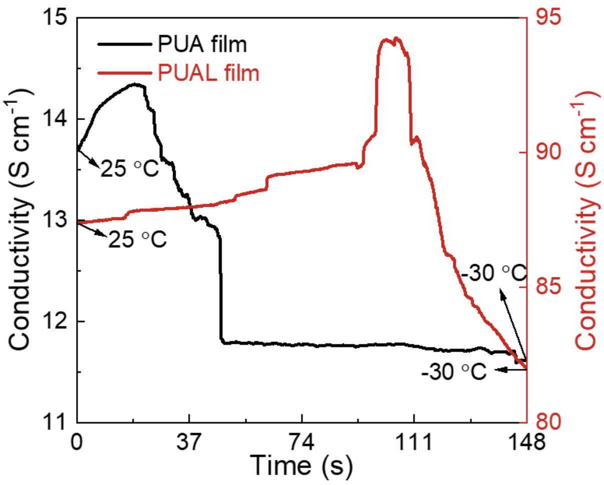

Supplementary Figure 28. Conductivity variation of PUA and PUAL films from 25 °C to -30 °C.

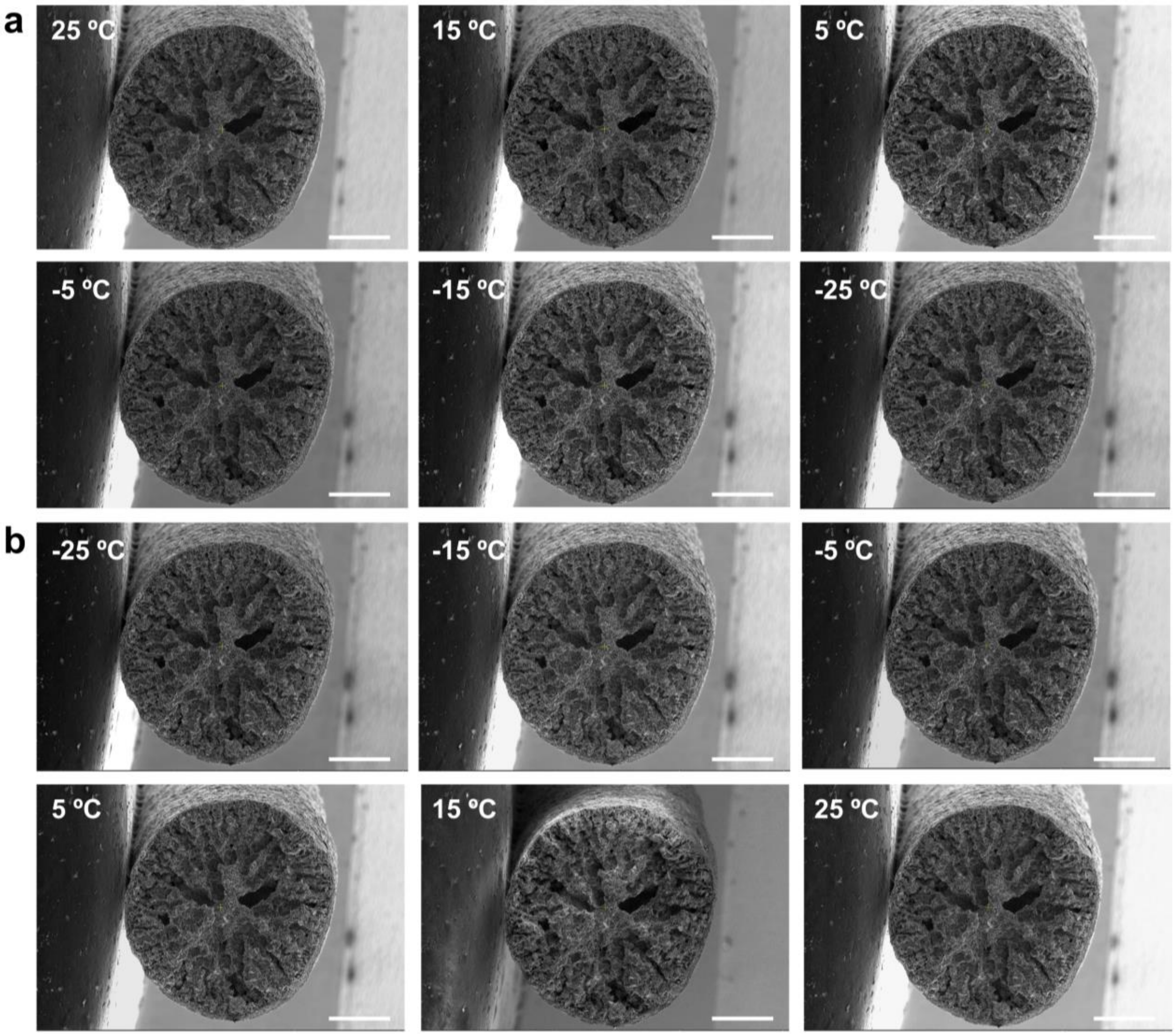

Supplementary Figure 29. The cross-sectional in-situ SEM images of the PUAL fiber from (**a**) 25 ℃ to -30 ℃ and (**b**) back to 25 ℃. Scale bar: 100 μm.

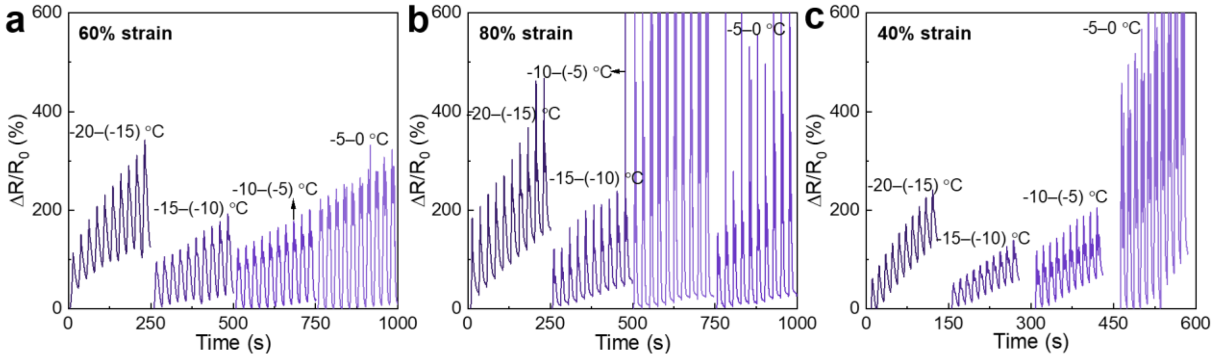

Supplementary Figure 30. Resistance change of the PUAL and PUA fibers under cyclic stretching at different strains and temperatures. a, Cyclic stretching of PUAL fiber with 60% strain from -20 ℃ to 0 ℃. **b**, Cyclic stretching of PUAL fiber with 80% strain from -20 ℃ to 0 ℃. **c**, Cyclic stretching of PUA fiber with 40% strain from -20 ℃ to 0 ℃. 20 cycles were tested for each condition, with the last 10 cycles plotted for clarity. Shades of purple from dark to light in **a**–**c** represent the temperature ranges of -20 °C to -15 °C, -15 °C to -10 °C, -10 °C to -5 °C, and -5 °C to 0 °C, respectively.

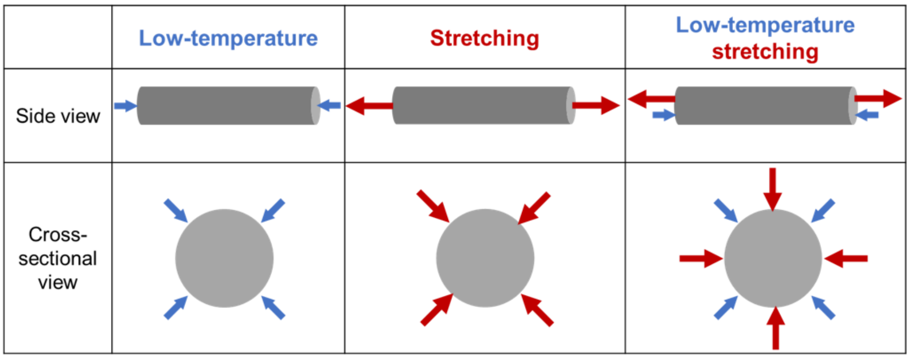

Supplementary Figure 31. Force analysis of PUAL fiber under low temperature and stretching conditions.

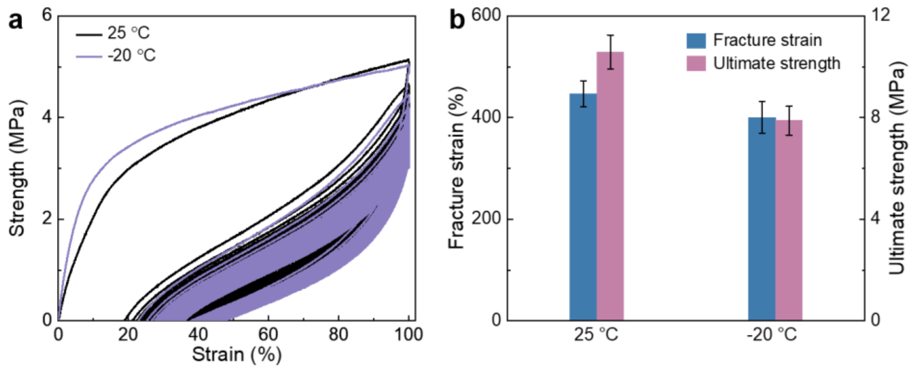

Supplementary Figure 32. Comparison of (**a**) tensile loading-unloading curves with 100% strain for 100 cycles and (**b**) fracture strain and ultimate strength of the PUAL fiber at 25 ℃ and after freezing at -20 ℃ for 30 minutes (five parallel samples were measured, and error bars represent the standard deviation of the mean).

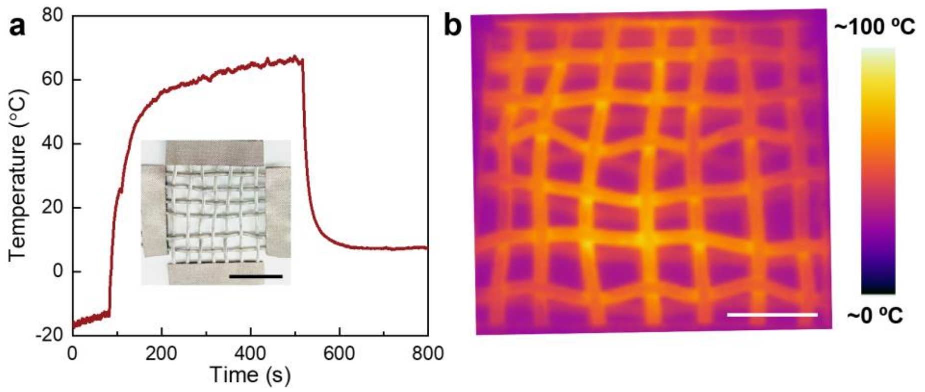

Supplementary Figure 33. Electro-heating performance of a fabric heater woven with PUAL fibers driven by 3 V voltage. a, Time-dependent surface temperature of PUAL fibers (inset: digital photo of the heater, scale bar: 2 cm). **b**, Infrared thermogram (scale bar:1 cm).

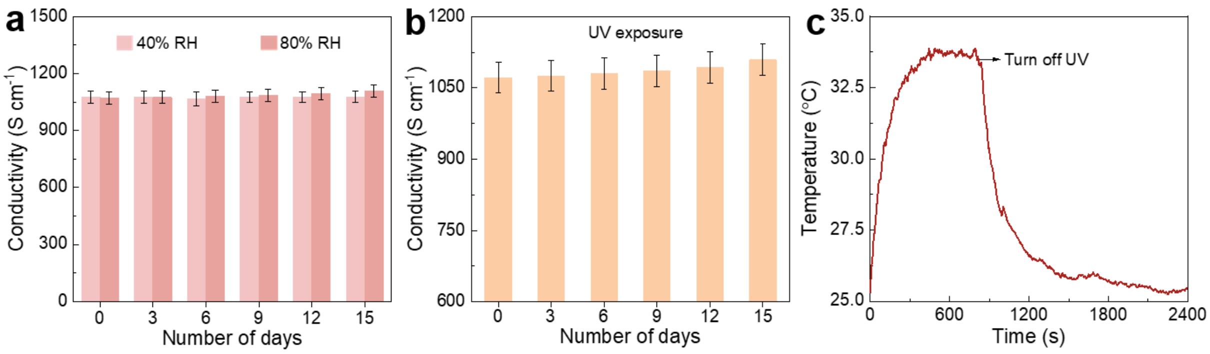

Supplementary Figure 34. Electrical durability of PUAL fibers under different conditions. a, The fiber's conductivity variation over 15 days under ambient conditions (40% humidity and 80% humidity) (five parallel samples were measured, and error bars represent the standard deviation of the mean). **b**, The fiber's conductivity variation over 15 days under ultraviolet (UV) exposure (10 W, 365 nm, distance of 12 cm, 7 hours every day) (five parallel samples were measured, and error bars represent the standard deviation of the mean). **c**, Temperature variation of the fiber during switching on and turning off the UV (10 W, 365 nm, distance of 12 cm). The slight increase in conductivity under UV exposure may be attributed to the temperature rise under UV.

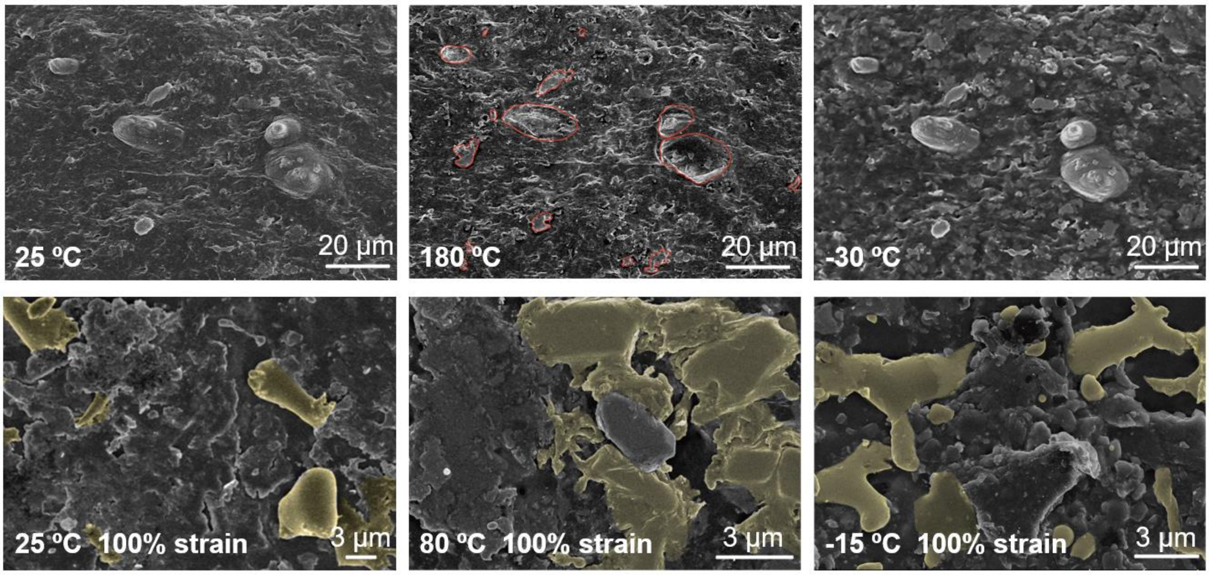

Supplementary Figure 35. SEM images of PUAL fiber under different temperatures, with or without tensile strain. The main deformed & ruptured LMMSs at 180 °C without tensile strain have been marked by red circles, and the main ruptured LMMSs under different temperatures and stretching conditions have been marked in bright yellow, indicating that LMMSs mainly rupture upon mechanical or thermal-mechanical stimulations.

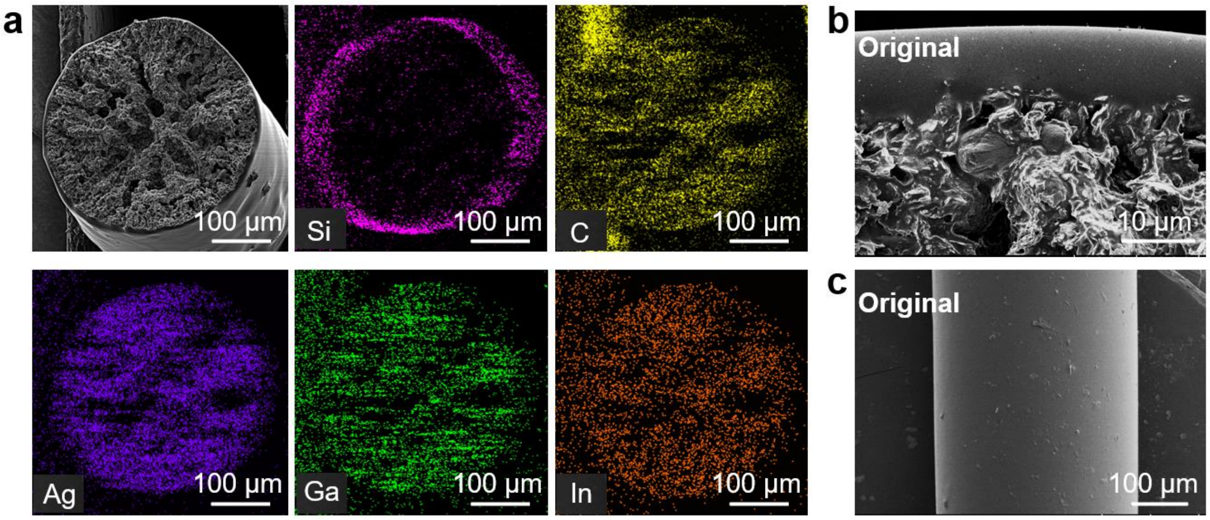

Supplementary Figure 36. SEM images and EDS mapping of PUAL fiber encapsulated with polydimethylsiloxane (PDMS). a, Cross-sectional SEM and EDS mapping. **b**, Enlarged cross-sectional SEM. **c**, Surface SEM. We encapsulated PUAL fibers using 50 wt% PDMS solution. The encapsulated fiber shows a smooth surface with a uniform PDMS layer of about 10 μm. The Ga and In signals at the left of the fiber are from the previous contamination of the stub.

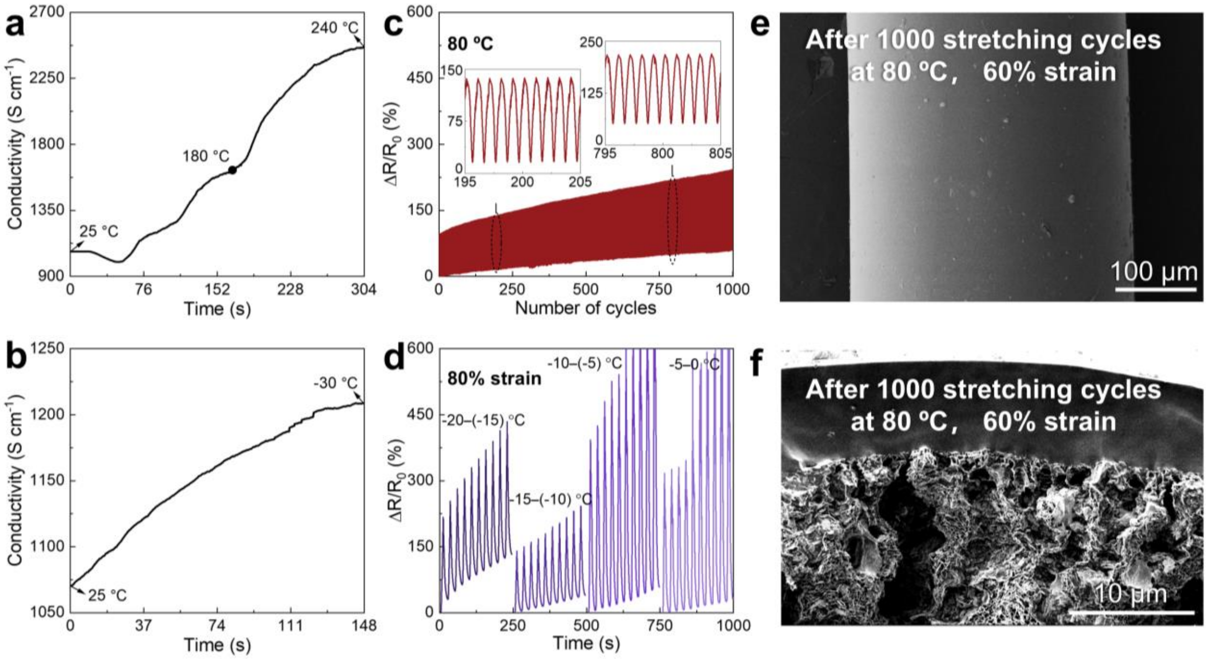

Supplementary Figure 37. Properties of PDMS-encapsulated PUAL fiber. a, Conductivity variation from 25 °C to 240 °C. **b**, Conductivity variation from 25 °C to -30 °C. **c**, Resistance changes under cyclic stretching with 60% strain at 80 °C over 1000 stretching cycles. **d**, Resistance changes under cyclic stretching with 80% strain from -20 °C to 0 °C. 20 cycles were tested for each temperature range, with the last 10 cycles for clear plotting. Shades of purple from dark to light in **d** represent the temperature ranges of -20 °C to -15 °C, -15 °C to -10 °C, -10 °C to -5 °C, and -5 °C to 0 °C, respectively. **e**, Surface morphology of the encapsulated fiber after cyclic stretching with 60% strain at 80°C over 1000 stretching cycles. **f**, Cross-sectional SEM image of the encapsulated fiber after cyclic stretching with 60% strain at 80 °C over 1000 stretching cycles. The encapsulation does not sacrifice the fiber's electrical properties. A similar conductivity-enhancement behavior as the original fiber was observed, which increases from 1070 S cm^{-1} (25 °C) to 1208 S cm^{-1} (-30 °C) and 1608 S cm^{-1} (180 °C) and 2463 S cm^{-1} (240 °C) under cooling and heating conditions (**a**, **b**), respectively. In addition, the encapsulated fiber can also be stretching-activated to improve its mechanoelectrical and thermoelectrical stability, maintaining stable resistance variation ($\Delta R/R_0$ of ~240% at 60% strain over 1000 stretching cycles, 80 °C, and ~240% at 80% strain, ~ -15 °C) (**c**, **d**), with similar trends align with the value without encapsulation. Satisfactory interfacial stability was observed on the fiber's cross-section after 1000 cycles of stretching deformation (**e**, **f**), indicating this encapsulation is an acceptable strategy for improving the fiber's electromechanical stability.

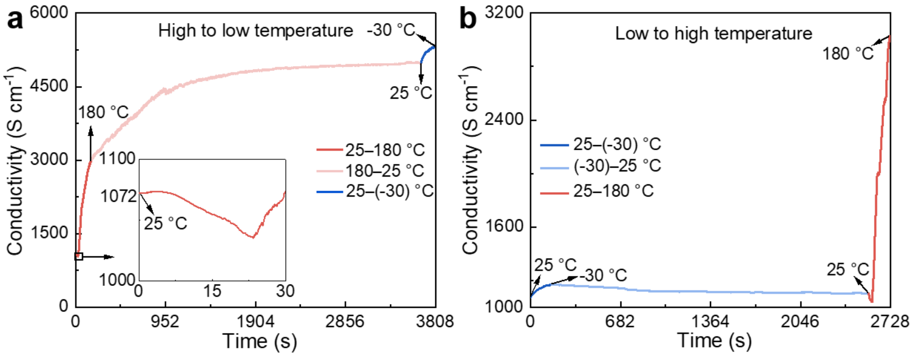

Supplementary Figure 38. Continuous conductivity variation when PUAL fiber is subjected to (**a**) a continuous heating-natural cooling-freezing process and (**b**) a continuous freezing-natural warming-heating process.

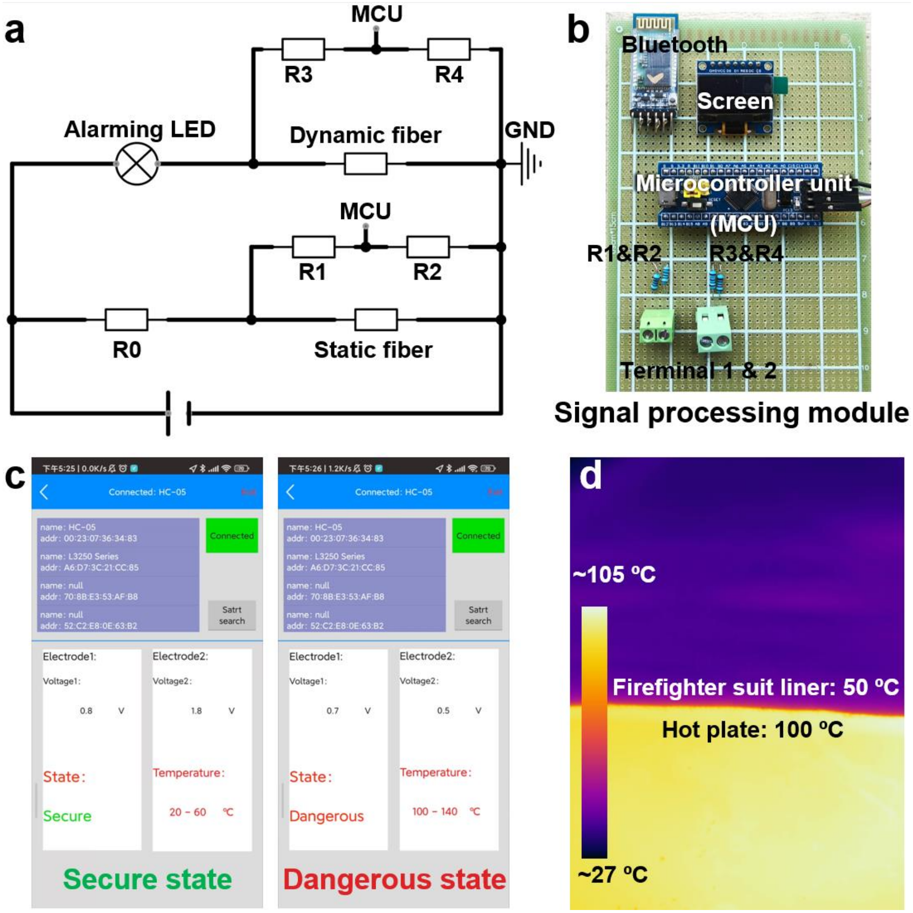

Supplementary Figure 39. High-temperature warning system design for temperature detection. a, Circuit diagram of the high-temperature warning system, where GND represents the ground. R1 = R2 = 1 kΩ, R3 = R4 = 10 kΩ. **b**, Digital photo of the circuit board. **c**, Screenshot of the Bluetooth APP of the wireless high-temperature warning system based on PUAL fibers. **d**, Temperature difference between the inner lining and the outside of the firefighter suit.

Supplementary Table 1. Diameter uniformity and electrical conductivity and mechanical properties across a 60 m-long PUAL fiber. 10 points across the 60 m-long PUAL fibers were measured, indicating the stable production process of this fiber.

Property	Mean value	Standard deviation
Diameter uniformity	350.2	16.3
Conductivity	1070.8	26
Fracture strain	451	25.6
Ultimate strength	10.6	0.7

Supplementary Table 2. Electrical conductivity, stretchability, and thermal stability trade-off of the PUAL fiber with other ECFs. Subject to the test conditions, the strain corresponding to -30 °C and 140 °C refers to the fracture strain of the PUAL fiber obtained after freezing or heating treatments for 30 minutes at the corresponding temperatures, respectively.

<table>
<tr><th>Material</th><th>Temperature (°C)</th><th>Conductivity (S cm^{-1})</th><th>Strain (%)</th><th>Ref.</th></tr>
<tr><td>PU/AgNWS/Carbon nanotube (CNT)</td><td>25</td><td>0.006</td><td>140</td><td>[6]</td></tr>
<tr><td>PU/Silver nanoparticle (AgNP)/Carbon black (CB)/AgNWs</td><td>25</td><td>0.25</td><td>400</td><td>[7]</td></tr>
<tr><td>PDMS/Au</td><td>25</td><td>8</td><td>100</td><td>[8]</td></tr>
<tr><td>Diketopyrrolopyrrole/$FeCl_3$</td><td>25</td><td>6</td><td>180</td><td>[9]</td></tr>
<tr><td>CNT</td><td>25</td><td>1000</td><td>300</td><td>[10]</td></tr>
<tr><td>Few-walled carbon nanotube (FWNT)/Poly(m-phenylene isophthalamide) (PMIA)</td><td>25</td><td>160.41</td><td>150</td><td>[11]</td></tr>
<tr><td rowspan="3">Aramid nanofibers (ANF)/MXene</td><td>25</td><td>3000</td><td>13</td><td rowspan="3">[12]</td></tr>
<tr><td>-196</td><td>3000</td><td>N/A</td></tr>
<tr><td>300</td><td>3000</td><td>N/A</td></tr>
<tr><td rowspan="3">TPU/AgFKs/LMMS</td><td>25</td><td>1070</td><td>450</td><td rowspan="3">This work</td></tr>
<tr><td>-30</td><td>1160</td><td>400</td></tr>
<tr><td>140</td><td>2025</td><td>390</td></tr>
</table>

Supplementary Table 3. Variation of PUAL fiber's cross-sectional area as the temperature changes from 25 °C to -30 °C, then back to 25 °C. The cross-sectional area was measured from in situ SEM images.

**Temperature (°C)**	**Cross-sectional area (10^4 μm^2)**
25	9.7157
20	9.6975
15	9.6775
10	9.6574
5	9.6520
0	9.6374
-5	9.6301
-10	9.6137
-15	9.6101
-20	9.5991
-25	9.5828
-30	9.5828
-25	9.5991
-20	9.6137
-15	9.6338
-10	9.6411
-5	9.6538
0	9.6684
5	9.6811
10	9.6884
15	9.7048
20	9.7085
25	9.7139

Supplementary Table 4. Relationship between the temperature and the dispensed voltage at both ends of the static PUAL fiber.

**Temperature (℃)**	$20 \leq T \leq 60$	$60 < T \leq 100$	$100 < T \leq 140$
**Voltage range (V)**	$V \geq 1.7$	$0.6 \leq V < 1.7$	$0.1 \leq V < 0.6$

Supplementary references

1. Yang, J., Su, Y., Song, G., Li, R. & Xiang, C. A new approach to predict heat stress and skin burn of firefighter under low-level thermal radiation. *Int. J. Therm. Sci.* **145**, 106021 (2019).
2. Choi, C., Schlenker, E., Ha, H., Cheong, J.Y. & Hwang, B. Versatile applications of silver nanowire-based electrodes and their impacts. *Micromachines-Basel* **14**, 562 (2023).
3. Wang, X. et al. Stretch-induced conductivity enhancement in highly conductive and tough hydrogels. *Adv. Mater*. **36**, 2313845 (2024).
4. Guo, H. et al. Highly anisotropic thermal conductivity of three-dimensional printed boron nitride-filled thermoplastic polyurethane composites: effects of size, orientation, viscosity, and voids. *ACS Appl. Mater. Interfaces* **14**, 14568-14578 (2022).
5. Kim, H.C., Kim, D., Lee, J.Y., Zhai, L. & Kim, J. Effect of wet spinning and stretching to enhance mechanical properties of cellulose nanofiber filament. *Int. J. Precis. Eng. Man-GT.* **6**, 567-575 (2019).
6. Ning, C. et al. Flexible and stretchable fiber-shaped triboelectric nanogenerators for biomechanical monitoring and human-interactive sensing. *Adv. Funct. Mater*. **31**, 2006679 (2021).
7. Choi, S. et al. Conductive hierarchical hairy fibers for highly sensitive, stretchable, and water-resistant multimodal gesture-distinguishable sensor, VR applications. *Adv. Funct. Mater.* **29**, 1905808 (2019).
8. Zhang, B. et al. Stretchable conductive fibers based on a cracking control strategy for wearable electronics. *Adv. Funct. Mater.* **28**, 1801683 (2018).
9. Zhao, Y. et al. Continuous melt-drawing of highly aligned flexible and stretchable semiconducting microfibers for organic electronics. *Adv. Funct. Mater.* **28**, 1705584 (2018).
10. Zhang, Y. et al. Flexible and stretchable lithium-ion batteries and supercapacitors based on electrically conducting carbon nanotube fiber springs. *Angew. Chem. Int. Ed.* **126**, 14792-14796 (2014).
11. Jiang, S. et al. Highly stretchable conductive fibers from few-walled carbon nanotubes coated on poly(m-phenylene isophthalamide) polymer core/shell structures. *ACS Nano* **9**, 10252-10257 (2015).
12. Liu, L.-X. et al. Super-tough and environmentally stable aramid. nanofiber@MXene coaxial fibers with outstanding electromagnetic interference shielding efficiency. *Nano-Micro Lett*. **14**, 111 (2022).
