# Supplementary material for: A temperature-adaptive component-dynamic-coordinated strategy for high-performance elastic conductive fibers
Source: Nat Commun. 2025 Jul 23;16:6785. doi: 10.1038/s41467-025-62140-y (PMC12287416; doi:10.1038/s41467-025-62140-y)
Supplement: Supplementary file 3 — Description of Additional Supplementary Files [file 41467_2025_62140_MOESM3_ESM.pdf]

Description of Additional Supplementary Files

File Name: Supplementary Movie 1

Description: ECG monitoring with woven PUAL fiber electrodes.

File Name: Supplementary Movie 2

Description: PUAL fiber NFC glove functioning in low and high temperature environments.

File Name: Supplementary Movie 3

Description: PUAL fiber integrated firefighting suit as a remote safety monitoring platform.
